# Initiation and Suppression of Crack Propagation during Magnesium Alloy Rolling

**DOI:** 10.3390/ma14185217

**Published:** 2021-09-10

**Authors:** Jing Tian, Quan-Xin Shi, Li-Xin Meng, Jia-Fei Deng, Wei Liang, Jin-Yao Ma

**Affiliations:** 1 College of Materials Science and Engineering, Taiyuan University of Technology, Taiyuan 030024, China; tianjing5678@163.com (J.T.); ajj20201010@163.com (Q.-X.S.); T1992417@163.com (L.-X.M.); tianmiaowang@163.com (J.-F.D.); 2 Shanxi Key Laboratory of Advanced Magnesium-Based Materials, Taiyuan University of Technology, Taiyuan 030024, China; 3 Instrumental Analysis Center, Taiyuan University of Technology, Taiyuan 030024, China

**Keywords:** texture, twinning deformation, dislocation, lattice distortion, shear

## Abstract

The conventional rolling of magnesium alloy with a single pass and large reduction will cause severe edge cracking. The sheet without cracks can be achieved by limited width rolling. The microstructure evolution of the sheet with cracks after conventional rolling and the sheet without cracks after limited width rolling is explored, and an effective mechanism for solving edge cracks is proposed. Conventional rolling can fully develop twin evolution due to high deformation, and three stages of twinning evolution can be observed and the secondary twins easily become the nucleation points of micro cracks, resulting in a large number of cracks propagating along the twin lamellae. Cracks terminate at dislocation accumulation because the accumulation of a large number of dislocations can hinder propagation. Dislocation shearing of twins to eliminate the high localization caused by twins and induce the tensile twins to weaken the basal surface texture provides an effective plastic deformation mechanism of crack inhibition, which is useful for expanding the engineering application of magnesium alloy rolled sheets.

## 1. Introduction

Magnesium alloy is the lightest metal structural material in actual engineering, with light specific gravity, high specific strength, excellent thermal and electrical conductivity [1,2,3,4,5,6], and the mechanical properties of rolled magnesium alloys are better than those of cast magnesium alloys. Therefore, magnesium alloy rolled sheets are widely used in automobiles, aerospace, and other fields. However, magnesium alloy has the defect of poor plastic deformation ability [2,7,8,9,10]. In the rolling process, it is easy to generate cracks at the edge of the magnesium alloy sheet during rolling and expand into the sheet. Especially when rolling in a single pass with large thickness reduction, serious edge cracks will occur, failing to roll. Cutting off the edge cracks will result in low material utilization and higher costs. This hinders the rolling of magnesium alloys under a single pass with a large reduction. Although rolling in multiple passes can obtain the magnesium alloy thin sheet with a large reduction thickness, the process is greatly increased. Thus, solving the problem of edge cracks in magnesium alloy rolling with large thickness reduction and improve its formability is particularly important.

The current research on the edge cracking problem in magnesium alloy rolling is as follows. In the process of magnesium alloy rolling, the uneven temperature distribution and material flow at the edge and the center lead to edge cracks [11]. Huang et al. found that excessively high or too low temperature in the rolling process of the sheet will cause severe edge cracking, and the increase of the reduction will lead to plastic deformation of the edge of the sheet. As the thickness reduction increases, the degree of edge damage also increases. M.R.G. Ferdowsi et al. [12] found that twinning and shear bands control the deformation process of AZ31 during rolling. Prefabricated edge crown before the rolling is an effective method to significantly reduce the edge cracking of Mg alloy sheets [13]. Jia et al. [14] analyzed the mechanisms and morphologies of edge crack during the axial compression process of AZ31 Mg alloy. Jing et al. [15] used width-limited to achieve no edge crack under rolling of magnesium alloy sheets under small thickness reductions.

In this paper, the magnesium alloy sheet is subjected to conventional rolling under a large thickness reduction in a single pass to obtain the sheet with crack, and the sheet without cracks is obtained by limited width rolling. By studying the microstructure evolution of the sheets with cracks after conventional rolling and without cracks after limited width rolling from the edge to the center of the sheet, the mechanism of crack propagation and inhibition in rolling of magnesium alloy is explained, which provides an effective mechanism for solving edge cracks in magnesium alloy rolling.

## 2. Material and Methods

**Figure 1:**
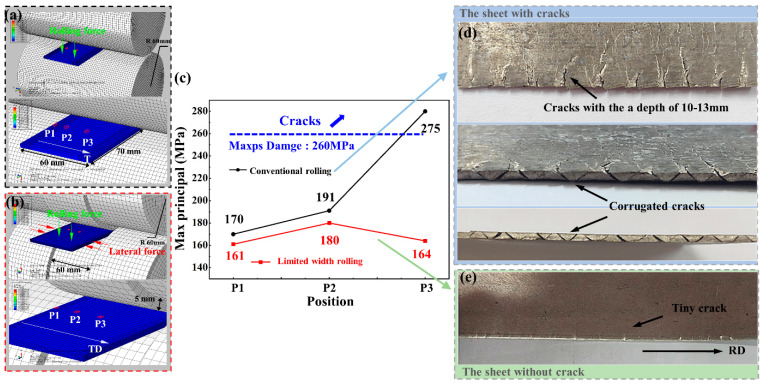
Schematic diagrams of rolls and ABAQUS model: (**a**) conventional rolling; (**b**) limited width rolling. (**c**) Max principal (Abs) of P1, P2, and P3 on the sheets at rolling time. (**d**,**e**) Edge crack situation of the sheets.

In this paper, a commercial magnesium alloy sheet (as-received sheet) with a size of 70 mm × 60 mm × 3 mm is subjected to conventional rolling and limited width rolling in a single pass at 673 K and rolled to 1.5 mm, which is equivalent to the thickness reduction of 50% to create severe edge cracks that cannot be solved by high-temperature rolling. The composition of the AZ31 magnesium alloy sheet used in this paper is shown in Table 1 and the rolling parameters for hot rolling of the magnesium alloys is shown in Table 2. The process is simulated by ABAQUS finite element software to explore the stress distribution from the edge to the center of the sheet during the rolling process. Figure 1a,b are schematic diagrams of conventional rolling and limited width rolling.

Edge cracks are generated along the transverse direction (TD). Samples are cut from P1, P2, and P3 on the rolled sheet as shown in Figure 1d,e to study the evolution of edge cracks. Position 1 (P1) is at the edge of the sheet, Position 2 (P2) is at the middle of the edge of the sheet and the center of the sheet and is 15 mm from the edge. Position 3 (P3) is at the center of the sheet and is 30 mm from the edge.

Microstructure characterization of the samples was performed at the plane consisting of the rolling direction (RD) and ND and analyzed by using an optical microscope (OM; Leica–2500M, Leica, Germany) and a field emission scanning electron microscope (SEM; TESCAN Mira 3, TESCAN, The Czech Republic) equipped with an electron backscatter diffraction (EBSD) system. These specimens were all grinding on 400, 800, 1200, 1500, 2000, and 3000 grit SIC paper. The OM specimens were mechanically polishing for 10 min and the EBSD specimens were electro-polishing at a voltage of 45 V and an electric current of 0.05 A for 45 s at a temperature of −20 °C. Macro texture consisting of RD and TD of P1, P2, and P3 of the rolled sheets was characterized by X-ray diffraction (XRD) using Cu Kα radiation on a RIGAKU Smart Lab SE at a scan rate of 5°/min and step size of 5°. The X-ray line profiles were measured using the same X-ray diffraction device at a wavelength of 0.154184 nm and a scan rate of 2°/min. The data from XRD is used to calculate the total dislocation density. Transmission electron microscopy (TEM, JEM-2100F, JEOL, Tokyo, Japan) was used to characterize the dislocations and the twins. The samples were prepared by electro-polishing and observation was conducted with an accelerating voltage of 200 kV.

The tensile specimens of 3 mm width and 25 mm length were cut from the sheets with cracks and without cracks after annealing for 473 K 6 h along the RD. The mechanical properties of the sheets with cracks and without cracks tested using a universal material testing machine (WDW-200, Kexin Test Instrument, Changchun, China) with a crosshead speed of 0.5 mm/min at room temperature.

## 3. Results and Discussion

### 3.1. Crack Propagation

**Figure 2:**
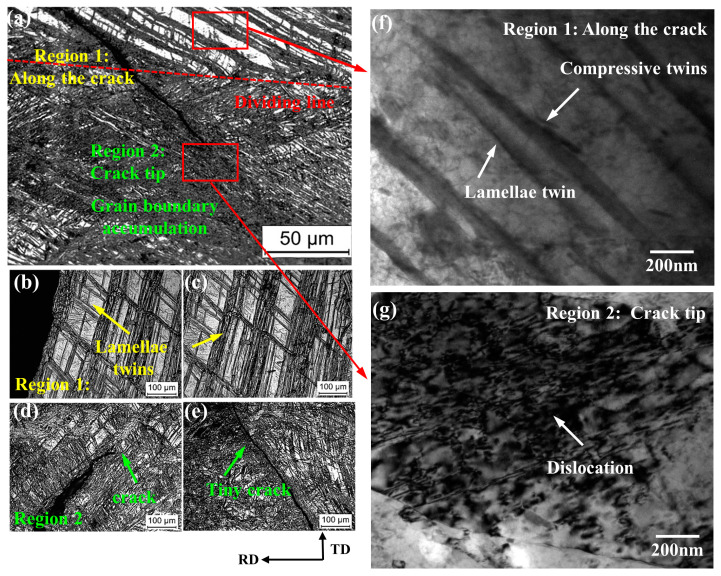
(**a**–**e**) Microstructure near the edge cracks of the sheet with cracks. TEM image showing the feature of lamellae twins in the (**f**) region 1 and (**g**) region 2.

Before further exploring the mechanism of suppressing edge cracks, first explore the microstructure near the crack under large thickness reduction. Figure 2 shows the microstructure near the crack. As shown in Figure 2a, there is a clear dividing line between region 1 and region 2. In region 1, the crack propagates from lamellae twins as shown in Figure 2b,c; in region 2, the crack tip ends at the accumulation of grain boundaries, and the structure around tiny crack as shown in Figure 2e is relatively uniform, compared with a large crack in Figure 2d.

Figure 2f,g is TEM image in region 1 and region 2, respectively. It is found that the twin lamellae in region 1 are typical compressive twin morphology, thin and straight. Besides, the accumulation of grain boundaries in region 2 is caused by high-density dislocations, which hinders the further propagation of cracks.

**Figure 3:**
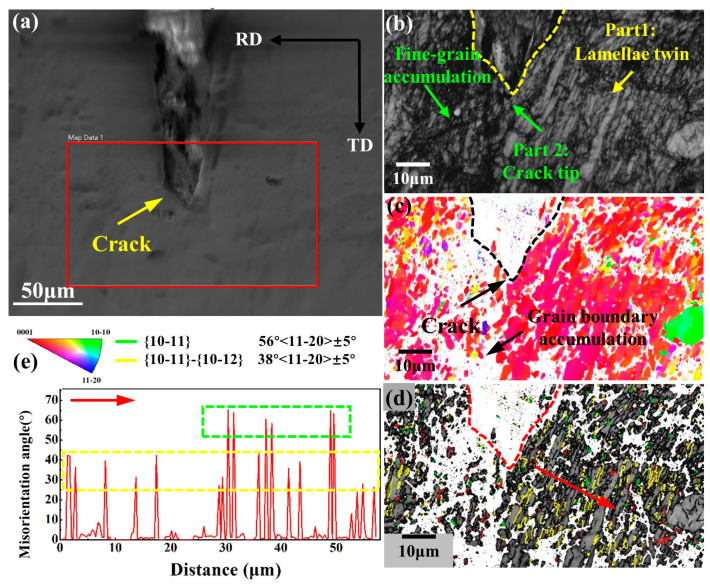
EBSD results at crack tip: (**a**) SEM image, (**b**) Band contrast, (**c**) inverse pole figure, and (**d**) Band contrast with three kinds of deformation twins in the red dotted line in Figure 3a. (**e**) Misorientation angle along red arrow in Figure 3d.

Figure 3 is EBSD results at the crack tip. Band contrast in Figure 3b is the same as the microstructure under OM in Figure 2a. Figure 3c is an IPF map and the crack tip cannot be analyzed due to excessive residual stress. Figure 3d,e show that the crack propagation direction is parallel to the secondary twin boundary and compressive twin boundary. There are many secondary twins and compressive twins near the crack in Figure 3e. This is because secondary twins cause a high degree of localization of the internal strain of the material, and these localized locations often develop into nucleation sites for micro cracks [16,17].

Therefore, conventional rolling under a large thickness reduction will produce a large number of compressive twins and secondary twins. The highly localized material strain caused by the secondary twins leads cracks to propagate along the twin boundaries, so the edge cracks of the magnesium alloy under large thickness reduction are serious. A large number of dislocations cause a large number of grain boundaries to accumulate to inhibit the further expansion of the crack, so the crack tip ends at high-density dislocation.

### 3.2. Difference of Crack Propagation and Inhibition

To obtain the mechanism of suppressing the edge cracks of magnesium alloys in a single pass with large thickness reduction, compare the difference between the sheet with cracks and without cracks from the edge to the center of the sheet.

#### 3.2.1. Stress Distribution

To study the stress distribution of the sheet with cracks and without cracks during rolling in a single pass with large thickness, ABAQUS finite element simulation software was used to simulate conventional rolling and limited width rolling and Abaqus/Explicit was chosen for the simulations.

The geometry and dimensions of the sheet and rolls are the same as those in the experiment, and the models are shown in Figure 1a,b. The material properties are listed in Table 3. A finer mesh is created at the edge of the sheet by creating partitions which enables finer mesh on the specific regions. For rollers and sheets, the same C3D8R elements were used. The model of rollers comprised 69,380 elements and the model of sheet comprised 13,330 elements.

When the stress or strain meets the initial critical damage criterion, then the degradation begins. For ductile damage of magnesium alloy, when the max principal is greater than the tensile strength of the sheet or the strain exceeds the displacement of the fracture, damage and cracks will occur. Figure 1c shows the stress along RD at P1, P2, and P3 of the sheets with cracks and without cracks. 

After conventional rolling, the stress at P1 on the edge of the sheet reaches 275 MPa, and the stress at the P2 is 191 MPa, which is very different from the stress on the center of the sheet (170 MPa). The sharp drop of stress results in the asynchronous extent between the central and the edge of the sheet and the stress along RD at P1 exceeds tensile strength, so that the crack extends along with TD to 10–13 mm deep, as shown in Figure 1d. Rolling in a single pass with a thickness reduction of 50% is a large deformation for the magnesium alloy thin sheet and magnesium alloy sheet exhibits poor plasticity under large deformation. A large number of cracks occur and the surface of the rolled sheet is relatively rough, as shown in Figure 1d. From the side of the sheet, that is, the surface formed by RD and ND, it can be found that many cracks are generated, forming a corrugated arrangement, which is very unfavorable for the formability of magnesium alloy. Thus, conventional rolling cannot achieve rolling in a single pass with a large thickness reduction of magnesium alloy.

The stress of the sheet with no crack is smaller than that of the sheet with cracks, and the stress distribution at the three positions of the sheet is more uniform, which is beneficial to the simultaneous extension of the sheet. It does not exceed the tensile strength of the sheet, so it will not cause cracking. As shown in Figure 1e, under the large deformation (the thickness reduction is 50%), the magnesium alloy sheet has almost no cracks and its surface is relatively smooth. The stress on the center of the sheet during conventional rolling (170 MPa) and the stress on the center of the sheet during limited width rolling (161 MPa) is almost the same. 

By changing the stress distribution of the sheet during the rolling process, the maximum principal stresses at the edges and the center of the sheet are both less than the tensile strength, which can effectively suppress the generation of edge cracks.

#### 3.2.2. Microstructure Evolution

**Figure 4:**
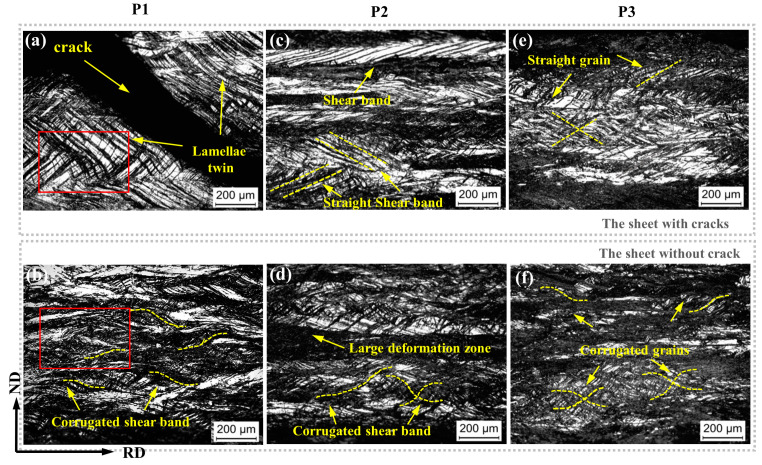
Microstructure evolution from the edge to the center of the sheets. The yellow arrows indicate twins, cracks, dislocations, and shear bands. (**a**) At P1, (**b**) at P2 and (**c**) at P3 of the sheet with cracks. (**d**) At P1, and (**e**) at P2 and (**f**) at P3 of the sheet without cracks.

Figure 4 exhibits microstructure evolution from the edge of the sheet to the center of the sheet. The first column is the microstructure of the sheet with cracks at P1, P2, and P3. The second column is the microstructure of the sheet without cracks at P1, P2, and P3. The grain orientation is marked with the yellow dotted line. The yellow arrows indicate twins, cracks, and shear bands.

There are many cracks generated on the sheet in Figure 1d. Figure 4a shows that the structure near the crack is the same as it in region 1 (Figure 2). The accumulation of the twin lamellae leads to uneven distribution of the structure, and the crack extends along the straight twin lamellae. The stress field of conventional rolling leads the twin straight and thin. A large number of lamellae twin boundaries lead to stress concentration at the edge of the sheet, resulting in crack propagation along the twin boundary. Limited width rolling makes the crystal grains corrugated, and makes the structure uniformly distributed, avoiding stress concentration and suppressing edge cracks.

At P2, that is, the middle part of the edge and the center of the sheet, the microstructure of the sheet with cracks show an uneven distribution. The shear band, which is a typically rolled microstructure, also leads to the uneven distribution of the microstructure to a certain extent. At P2 of the sheet without crack, there are a large number of corrugated twins and shear bands, leading to the accumulation of grain boundaries.

At the center of the sheet, there is still a slight difference between the sheet with cracks and without cracks. The sheet without cracks still maintains corrugated microstructure, while that of the sheet with cracks is still straight. The study found that, from the edge to the center of the sheet, the difference in the structure of the sheet without cracks and with cracks gradually decreases.

Greater deformation can homogenize the microstructure and leading the grain to change from straight to corrugated, which mainly acts on the edges of the sheet, from the edge to the center of the sheet, it gradually reduced.

The sheet without cracks produces greater plastic deformation during rolling in a single pass with large thickness reduction, resulting in a more uniform distribution structure. At the same time, twins and shear bands changed from straight to corrugated, and the edge of the sheet was most affected.

#### 3.2.3. Lattice Distortion

**Figure 5:**
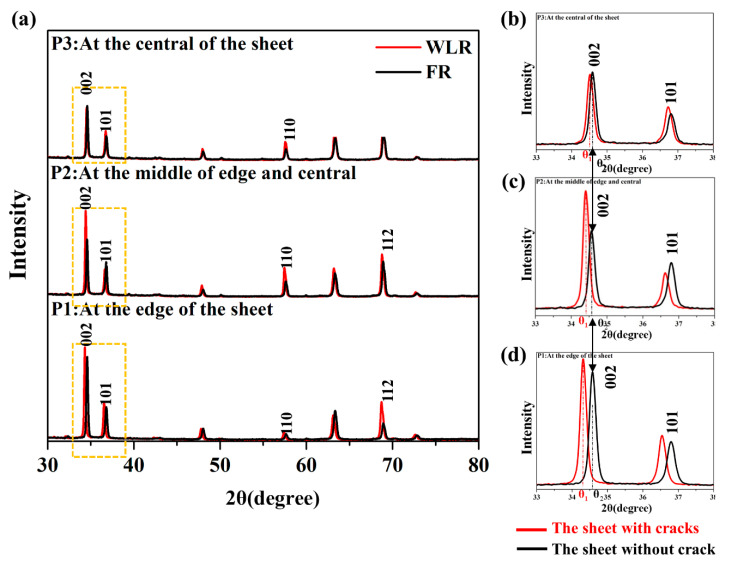
XRD spectra of the sheets without cracks and with cracks. (**a**) XRD spectra of the samples cut from P1, P2, and P3 on the sheets. (**b**–**d**) XRD spectra showing the marked yellow dotted line in Figure 5a.

Figure 5 is XRD spectra of the samples of the sheet with cracks and without cracks. Figure 5a shows XRD spectra of the samples cut from P1, P2, and P3 of the sheet with cracks and without cracks, the red line represents the samples of the sheet without cracks and the black line shows the samples of the sheet with cracks. Figure 5b–d are XRD spectra showing marked yellow dotted lines in Figure 5a. 2Theta of the peaks corresponding to the (002) basal of the sheets with cracks and without cracks is represented by θ_1_ and θ_2_, respectively. For 2Theta corresponding to the diffraction peaks of the same crystal plane, it is different for the samples and 2Theta of the sheet without a crack (θ_1_) is significantly smaller than that of the sheet with cracks (θ_2_). Besides, from the center of the sheet to the edge of the sheet, the position of the diffraction peak of the sheet without cracks is farther away from the sheet with cracks. The 2Theta of the sheet with cracks (θ_2_) is unchanged from the edge to the center, as shown by the black double arrow in Figure 5.

As a kind of line defect, dislocations cause the regular arrangement of atoms to be disturbed and destroyed, causing some atoms in the crystal to deviate from the normal position, and the atoms in the crystal are arranged in disorder. As a result, the crystal lattice expands, the distance between the crystal planes increases, and the 2Theta of the diffraction peaks decreases. Thurs, the smaller 2Theta, the greater the lattice distortion here. The deviation between the sheet without a crack (θ_1_) and the sheet with cracks (θ_2_) has resulted from crystal lattice distortion caused by dislocation. The 2Theta of the sheet with cracks (θ2) is unchanged, that is, the sheet with cracks basically has no lattice distortion during conventional rolling, and the deformation can be released in the form of cracks.

In order to make the sheet free of cracks, a large amount of sliding movement is required to coordinate the plastic deformation, resulting in the accumulation of various types of dislocations in the rolled sheet, leading the lattice distortion. θ_1_ is the smallest at the edge of the sheet and the largest lattice distortion exists here, so there is the largest deformation. From the edge to the center, the deviation between θ_1_ and θ_2_ is reduced, and the lattice distortion is gradually weakened, so it mainly acts on the edges.

**Figure 6:**
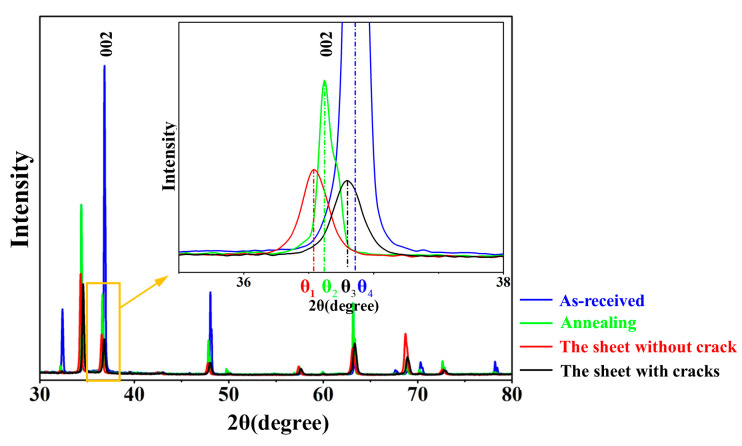
XRD spectra of the samples cut from the as-received sheet, the sheet without crack, the sheet with cracks, and the sheet without cracks after annealing for 180 °C, 2 h were compared.

To further verify that the deviation of the diffraction peaks is due to crystal lattice distortion, the positions of diffraction peak of the samples cut from the as-received sheet, the sheet with cracks, the sheet without crack, and the sheet without cracks after annealing for 180 °C, 2 h were compared, as shown in Figure 6. The as-received sheet has the largest diffraction angle (θ_4_), which does not produce lattice distortion. Annealing can reduce the lattice distortion. the sheet without cracks after annealing for 180 °C, 2 h (θ_3_) is between the sheet without a crack (θ_1_) and the as-received sheet (θ_4_). Therefore, the greater the lattice distortion, the greater the deviation of the diffraction peaks.

The large deformation energy of the sheet without cracks during rolling in a single pass with large thickness reduction is not released in the form of cracks, and all acts on the structure, so it will cause the generation of lattice distortion. Among them, the deformation of the edge is the largest, which produces the largest lattice distortion.

#### 3.2.4. Dislocation

Dislocation slip, as the dominant deformation mechanism of rolling in a single pass with a large thickness reduction of magnesium alloys, requires further research. First, a qualitative analysis of dislocations is carried. 

Combining the slip of HCP crystal and XRD data in Figure 5a, the sheets with cracks and without cracks had large deformation and high rolling temperature, leading to the activation of various types of slip systems to coordinate the deformation. Generally speaking, the half maxima of the diffraction peak has the following relationship with the dislocation density

(1) $$\mathrm{FWHM}\propto \sqrt{\rho }$$

In the formula, FWHM is full width at half maxima of the diffraction peak, ρ is the dislocation density.

According to Formula (1), the dislocation density is proportional to FWHM. the dislocation density corresponding to each slip plane of the rolled sheet without cracks is greater than it of the rolled sheet with cracks. 

The quantitative calculation of dislocations is as follows. Since both strain and grain size will cause the diffraction peak to broaden, it can be distinguished by the Williamson-Hall Equation [18]

(2) $$\frac{∆2\theta \cdot \cos\theta }{\lambda }=\frac{0.9}{D}+2\varepsilon \frac{\sin\theta }{\lambda }$$

In the formula, ∆2θ is full width at half maxima of the diffraction peak (FWHM), θ is the diffraction angle, λ is the wavelength of the X-ray at 0.154814 nm, and ε is the strain due to mechanical deformation, D is the grain size. 

**Figure 7:**
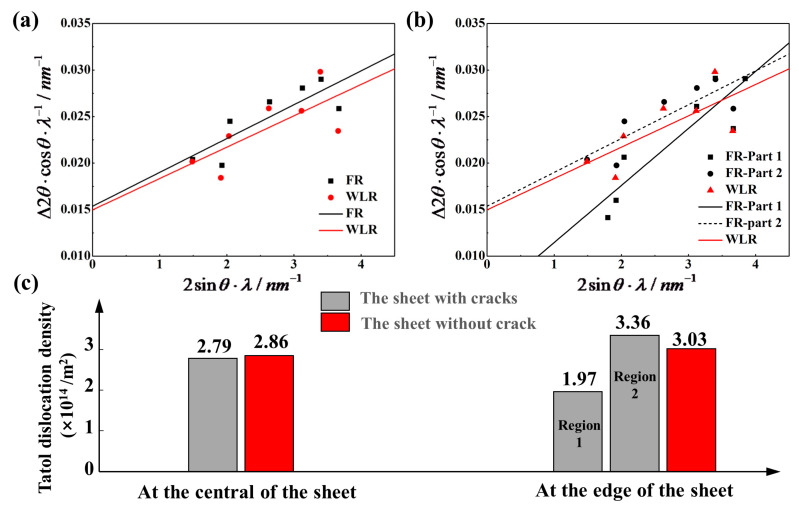
(**a**,**b**) Fit curve result of the sheets with cracks and without cracks. (**c**) Total dislocation density of different parts in the sheets without cracks and with cracks.

By measuring θ and Δ2θ of each diffraction peak, plotting with $\frac{\sin\theta }{\lambda }$ as the abscissa and $\frac{∆2\theta \cdot \cos\theta }{\lambda }$ as the ordinate, the slope of the fitted straight line is 2ε, and the intercept is $\frac{0.9}{D}$. The strain ε and the grain size D can be calculated from the relationship between $\frac{\sin\theta }{\lambda }$ and $\frac{∆2\theta \cdot \cos\theta }{\lambda }$, as shown in Figure 7a,b. The total dislocation density caused by plastic deformation can be calculated by the following formula

(3) $$\rho =\frac{2\sqrt{3}\varepsilon }{\mathrm{Db}}$$

where ε and D are the strain and grain size calculated by Williamson–Hall equation, respectively, b is the Persian vector of Mg alloy with a value of 0.32 nm [19,20]. Figure 7a,b are the fitted curve to calculate total dislocation density in different parts of the sheet with cracks and without cracks. The calculation results of the total dislocation density are shown in Figure 7c. 

At the center of the sheet, the total dislocation density of the sheet without cracks is the same as that of the sheet with cracks, and the dislocation density of the sheet without cracks is slightly higher than that of the sheet with cracks. This is because the lateral pressure of the sheet without cracks also has a slight influence on the center of the sheet. At the edge of the sheet, the dislocation density of the sample after the sheet without cracks is higher, and the dislocation density of the sample after the sheet with cracks is polarized. The crack propagates along with the lamellae twin of the edge of the sheet marked region 1 in Figure 2, where the dislocation density is extremely low, and at the crack termination marked region 2 in Figure 2, the dislocation density suddenly increases due to the accumulation of grain boundaries to prevent further crack propagation.

The complex stress field of the sheet without cracks during rolling makes the edge of the sheet bear greater plastic deformation, and no cracks generate to release energy, so the large deformation can activate various types of dislocations, resulting in the accumulation of high-density dislocations and the activation of <a + c> slip system to coordinate the strain of the c-axis have a positive effect on improving the formability of the magnesium alloy [21,22].

In summary, the maximum principal stresses at the edge and center of the sheet without cracks are less than the tensile strength, so edge cracks will not occur. The sheet without cracks bears greater plastic deformation. Large deformation energy can activate various types of slip systems, leading to the accumulation of high-density dislocations, leading to lattice distortion. The structure distribution of the sheet without cracks is more uniform and grains change from straight to corrugated. 

### 3.3. Plastic Deformation Mechanism of Crack Inhibition

#### 3.3.1. Interaction of Twinning and Slip

According to the difference of the rolled sheet from the edge to the center, it is found that the greatest impact on the microstructure is the edge of the sheet, so further research is carried out on the microstructure of the edge of the sheet to explore the plastic deformation mechanism of crack inhibition.

**Figure 8:**
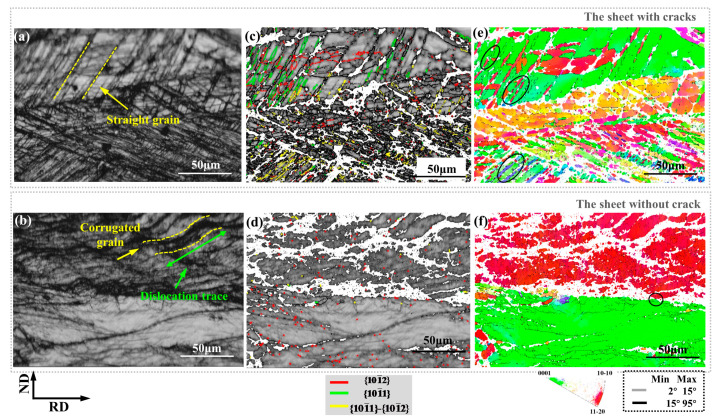
EBSD results of the edge of the sheet with cracks and without cracks. (**a**,**b**) Band contrast. (**c**,**d**) Band contrast with three kinds of twins. (**e**,**f**) Inverse pole figures.

Figure 8 is the EBSD result of the rolled sheet. Figure 8a,b are maps of the microstructure at the edge of the sheet, which are the same as the microstructure under OM in Figure 4a,b. At the edge of the sheet with cracks near the crack, it presents lamellae twins in Figure 4a and Figure 8a, and at the edge of the sheet without crack, Figure 4b and Figure 8b present corrugated grains and corrugated deformation bands.

**Figure 9:**
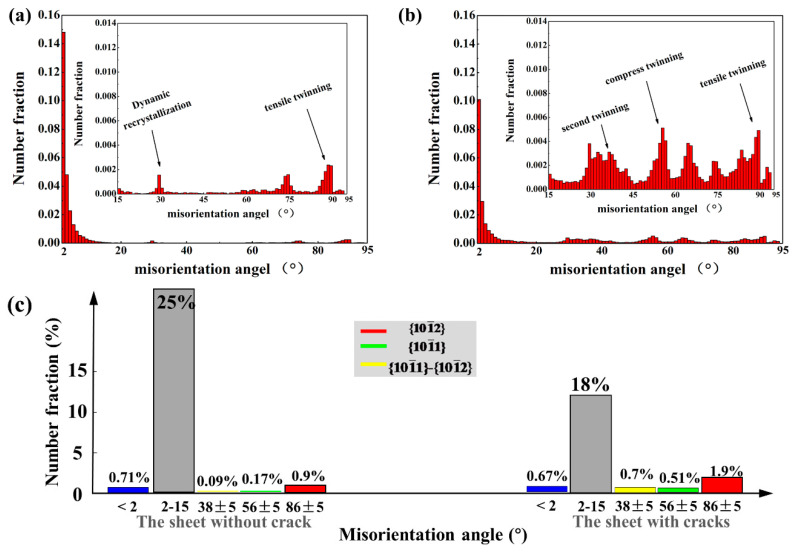
Misorientation angle of (**a**) the sheet with cracks and (**b**) the sheet without cracks. (**c**) Comparison of contents of sub-grain boundaries and other three kinds of twin boundaries.

There are many kinds of twins in Figure 8c. On the contrary, the twins in Figure 8d are not formed tensile twins. Figure 9a,b are corresponding misorientation angles. The sheet with cracks has obvious peaks of secondary, compressive, and tensile twins. However, there is almost no peak of twin in the sheet without cracks. The grain boundaries of small misorientation angle and sub-grain boundaries in Figure 9a account for a larger proportion, because the sheet without cracks bears greater deformation, causing dislocations to accumulate. Yet the sub-grain boundaries in the sheet with cracks are less than the sheet without cracks, that is, the dislocation is relatively less, and the peak of the twin is larger, so the twins play a certain role in the sheet with cracks under large deformation.

**Figure 10:**
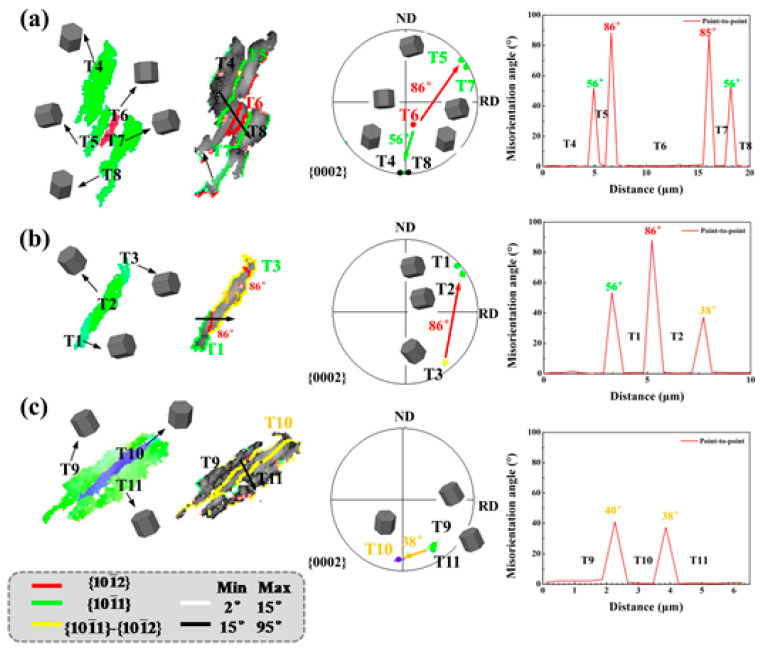
The twinning development of the sheet with cracks. Typical EBSD results of magnified view of the region marked with black circle in Figure 8e. (**a**) tensile twins and compressive twins. (**b**,**c**) double twins.

Figure 10 is an enlarged part of the black circle in Figure 8e. It shows the three stages of twinning development in the sheet with cracks: (1) independent tensile and compressive twins; (2) the process of transforming compressive twins to secondary twins; (3) compressive twins fully develop into secondary twins. Figure 8a shows the independent {10–12} tensile and {10–11} compressive twins. The relationship with the matrix is shown in the figure, consistent with the theoretical angle. Figure 10b shows the relationship between {10–11} compressive twin and {10–11} {10–12} secondary twin, in which some of the compressive twins develop into secondary twins. Secondary twins are tensile twins formed inside compressive twins, which are the further development of compressive twins. Compression twin is obtained by rotating the matrix 56° around the axis <11–20>. Then rotated 86° around the axis <11–20> to generate tensile twins, which is equivalent to rotating 38° around <11–20> [23,24,25,26]. The further twinning development will cause the compressive twins to fully develop into secondary twins. The mature development of twinning is still unable to coordinate the large deformation under rolling in a single-pass with large thickness reduction, and the secondary twins will become the nucleation point of micro-cracks, and the high localization of material strain leads to the generation of cracks.

**Figure 11:**
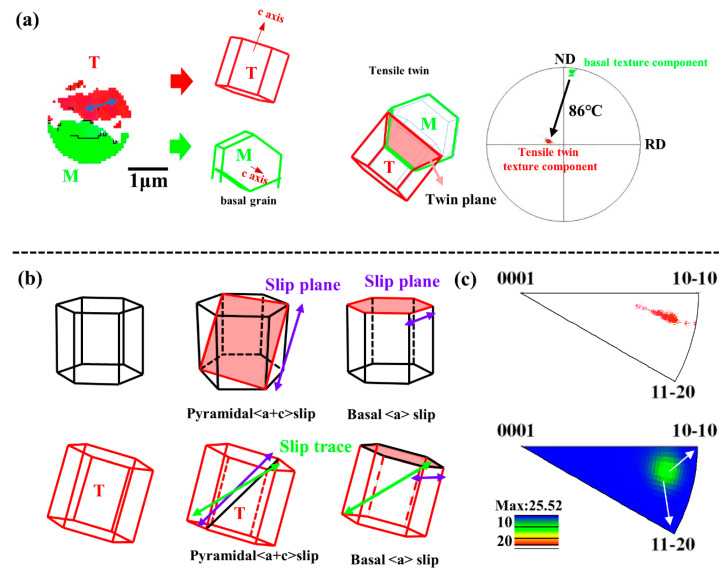
Plastic deformation mechanism of crack inhibition: (**a**) Twinning with an enlarged black circle in Figure 8f. (**b**) slip system. Purple arrows represent slip direction and green arrow represents slip plane trace, which is the same as the dislocation trace in Figure 8b. (**c**) In-grain misorientation axis (IGMA) analysis of T in Figure 11a.

Figure 11a is an enlargement of the black circle part of Figure 8f, showing the twinning plastic deformation mechanism. The corrugated grain T is formed by the grain M with basal texture rotated 86° around <0–22–1> and the red dot on the grain T in Figure 8c, indicating that there are {10–12} tensile twins. Therefore, the corrugated crystal grains are tensile twins, which cause the basal to deflect by 86°, thereby greatly weakening the basal texture. As shown in Figure 8c, the corrugated grains cannot be analyzed completely, which is due to a large number of dislocations that lead to excessive residual stress. The green arrow represents the slip plane trace [27]. Figure 11b shows the slip system of the tensile twin T, and the purple arrow indicates the slip direction. The slip trace is the co-activation of pyramidal <a + c> slip and basal <a> slip, which is consistent with the results obtained in IGMA [28]. 

**Figure 12:**
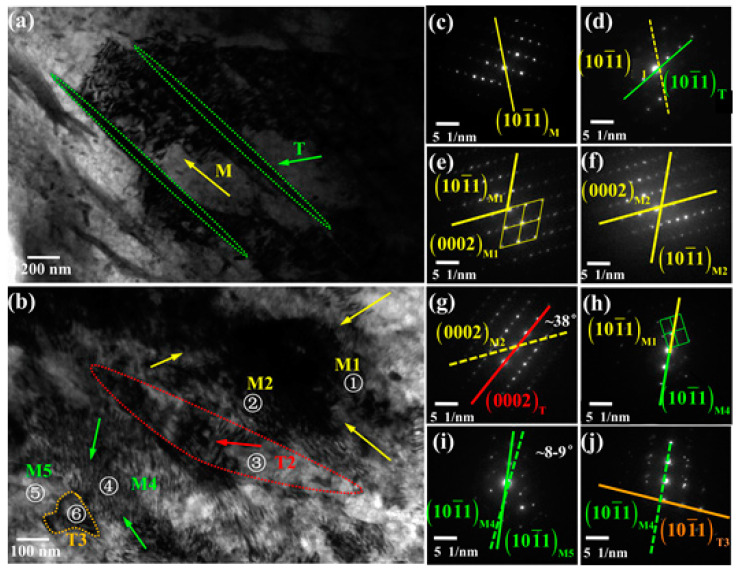
(**a**,**b**) TEM result of the sheets with cracks and without cracks. The electron beam is nearly parallel to the [−1010] zone axis. The arrow shows the dislocations of different regions. (**c**,**d**) The diffraction patterns taken from M and T in Figure 12a. (**e**–**j**) The diffraction patterns taken from the region 1, 2, 3, 4, 5, and 6.

Figure 12 is the TEM result. Figure 12a,b are TEM images showing the feature of deformation twins and dislocations near the edge of the sheet with cracks and the sheet without cracks. The outlines of twins are denoted by dotted lines. The twins are denoted by T, the matrix is marked by Mi (i = 1, 2, 4, 5). The electron beam is nearly parallel to the <2–1–10> axis. The arrow shows the dislocations of different regions.

The diffraction pattern in Figure 12c,d is taken from M and T in Figure 12a. It shows that compressive twins and a large number of dislocations are produced on the sheet with cracks.

The diffraction patterns of Figure 12e,j are respectively taken from regions 1–6 in Figure 12b. Region 1, 2, 4, and 5 represent different positions of the matrix, region 3 represents T2, and region 6 represents T3. The direction of <10–11> in Figure 12e,h is basically the same, but the grid is completely different, indicating that M4 has rotated around the axis <10–11>, which is caused by high-density dislocations. The deviation in Figure 12i may be caused by the inconsistency of the dislocation density at M4 and M5. Obviously, the matrix has also undergone huge deformation and a large amount of dislocation accumulation. 

The misorientation angle between M2 and T2 is 38°, which is very close to the theoretical value of the secondary twin (38°). That is, T2 is a secondary twin. The misorientation angle between M4 and T3 is 86°. However, T2 and T3 are not complete twin morphology. This is due to the large deformation of the sheet without cracks, and there is a high density of dislocations inside and outside the secondary twins, so the secondary twins also tend to be destroyed by dislocations.

In the sheet without cracks in a single pass with large thickness reduction, the sheared tensile twins and secondary twins are easily observed. The sheet without cracks was compressed along the transverse direction under rolling, that is, tensile twins can be prefabricated by compressing in the direction perpendicular to the c axis. At the same time, the large deformation leads to the mature development of twins, so the compressive twins further develop into secondary twins [29,30,31]. Compressive twins are rarely observed in the sheet without cracks due to two reasons: (1) Compressive twins almost fully develop into secondary twins under large deformation [32]. (2) Compressive twins are narrow elongated strips, even if some compressive twins are not maturely developed into secondary twins and are easily shear by large dislocations. 

#### 3.3.2. Weaken Texture

**Figure 13:**
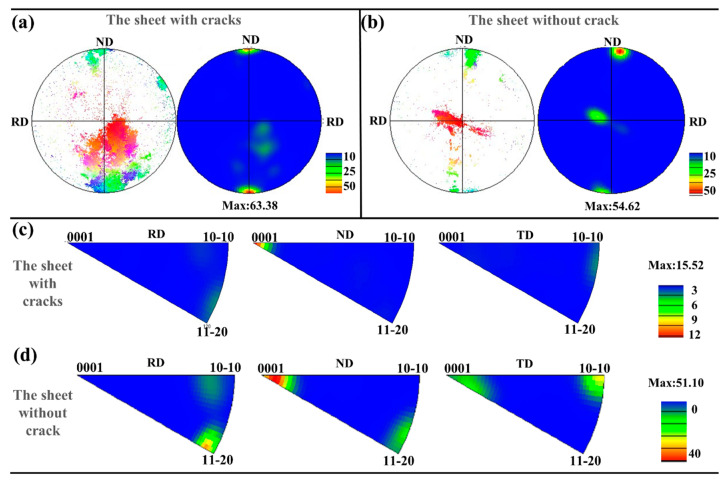
Pole figures of (**a**) the sheet with cracks and (**b**) the sheet without cracks. Inverse pole figures of (**c**) the sheet with cracks and (**d**) the sheet without cracks.

Figure 13a,b are the polar figures of the sheet with cracks and without cracks. It is found that the basal texture is weakened due to the deflection of the twins, and the basal can be deflected by 86° due to the tensile twins, so the effect to weaken the basal texture of the basal is more obvious. As shown in Figure 13c,d, ND∥ <0001>, so it forms a typical basal texture. RD is parallel to <11–20>and TD is parallel to <10–10>in a part of grains, as shown in Figure 13d.

**Figure 14:**
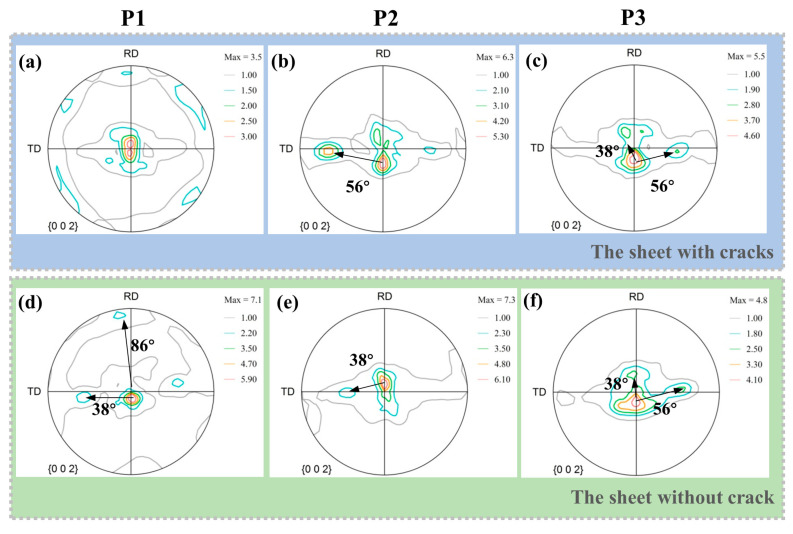
Macrotexture of the sheet with cracks and without crack: (**a**,**b**) at the edge of the sheet. (**c**,**d**) at the center of sheet. (**e**,**f**) at the middle between the edge and the center of sheet.

Texture measured by EBSD is the results analyzed in a relatively small area. In order to make the obtained results more representative, XRD was used to further explore the macrotexture of the edges and the center of the sheet without cracks and with cracks. The results are shown in Figure 14.

At the center of the sheet, the sheet with cracks and the sheet without cracks are fully rolled, resulting in a large number of secondary twins and compressive twins, so texture components are generated at 38° and 56°. The distribution of the basal texture component is roughly the same. At the edge of the sheet, the sheet without cracks performs more tensile twins, which have a texture component at 86°. The sheet with cracks produces a large number of secondary twins and produces texture components at 38°. Tensile twinning can make the basal deflection by 86°, so the effect of weakening basal texture is more obvious on the sheet without cracks.

#### 3.3.3. Soften Orientation

**Figure 15:**
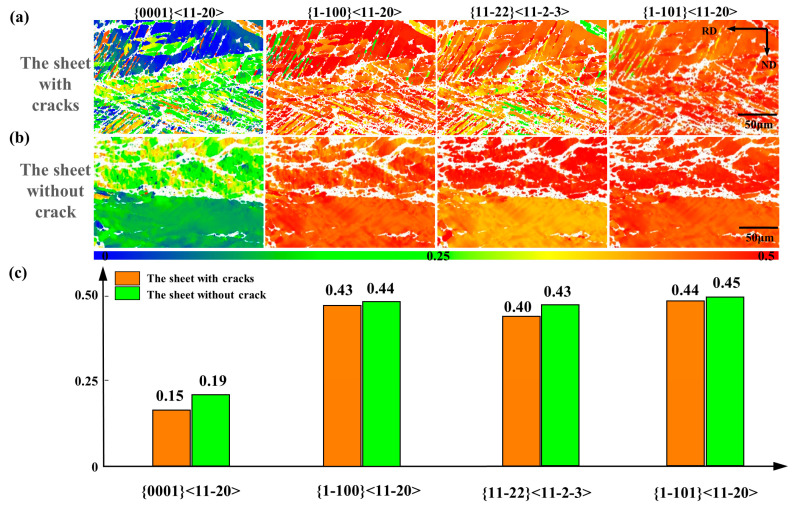
(**a**,**b**) Schmidt factor distribution and (**c**) average Schmidt factor value of the sheet with cracks and without cracks.

Figure 15 shows the comparison of Schmidt factor distribution and average Schmidt factor value of the sheet with cracks and without cracks. The loading direction is RD. 

As shown in Figure 15a, the Schmidt factor of the non-basal slip of the compressive twins is very low, so the edge of the sheet with cracks is in ‘hard’ orientation. The ability of the structure to coordinate deformation under a ‘hard’ orientation is very poor, and large plastic deformation cannot occur, so edge cracks occur and the deformation energy can be released. The corrugated tensile twins in Figure 15b, the characteristic structure of the sheet without crack, has a larger Schmidt factor value, especially the basal (0001) <11–20> slip and Pyramidal (11–22) <11–2–3> slip. Therefore, tensile twins of the sheet without cracks under transverse compression have a ‘soft’ orientation, which is more conducive to strain coordination [33,34]. The edge of the sheet without cracks is in a ‘soft’ orientation and can bear greater plastic deformation. No matter which slip systems, the average Schmidt factor of the sheet without cracks is higher than the sheet with cracks as shown in Figure 15c, so it has a stronger ability to coordinate deformation, especially (11–22) <11–2–3> slip system, which can coordinate c-axis. It is beneficial to improve the formability of the sheet and suppress the occurrence of cracks.

Tensile twins deflect the basal by 86°, effectively weakening basal, and the Schmidt factor of the corrugated tensile twins is large, and the grain orientation changes from hard orientation to soft orientation, which is beneficial to improve the plasticity of the sheet. The interaction of twinning and slip can make the sheet bear greater plastic deformation, and the edge of the sheet is in a soft orientation, which can better coordinate the strain, thereby effectively suppressing the edge cracks.

The plastic deformation of magnesium alloys mainly relies on twinning and slip, and slip is particularly important in large deformations. The sheet without cracks is transversely compressed, that is, the compression perpendicular to the c-axis of the grains produces corrugated tensile twins. The tensile twins deflect the basal by 86°, effectively weakening the basal texture, and the corrugated tensile twins have a high Schmidt factor, which is beneficial to coordinated deformation. Various types of slip are activated, especially <a + c> slip which can coordinate the strain of the c-axis. Dislocations are distributed in the matrix and the inside and outside of the twins, causing the twins to be sheared, therefore the twins have an incomplete morphology. The secondary twins are sheared and uniform the structure which avoids the highly localized strain of the material and cannot develop into micro cracks. The interaction of twinning and slip can make the sheet bear greater plastic deformation, and the edge of the sheet is in ‘soft’ orientation, which can better coordinate the strain, thereby effectively suppressing the edge cracking.

### 3.4. Contribution of Crack Inhibition

The sheet without cracks is not only beneficial to improve the forming rate of the rolled sheet, but also helps to improve the mechanical properties of the sheet.

**Figure 16:**
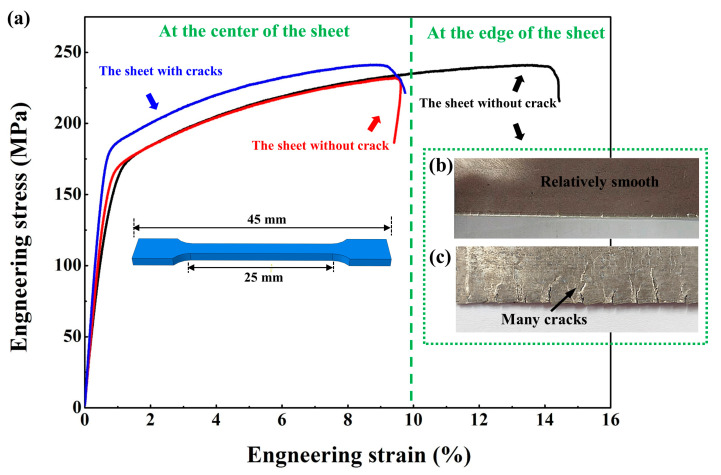
(**a**) Engineering stress–strain curve under tension of AZ31 rolled sheet at 50% thickness reduction after annealing for 473 K 6 h. (**b**,**c**) Edge crack situation.

Figure 16a is the engineering stress–strain curve. The blue line is the mechanical properties of the edge of the sheet without cracks. Edge cracks are serious and the mechanical properties at the edge of the sheet with cracks are extremely poor, so the tensile test cannot be carried out. The red line and the black line respectively represent the mechanical properties of the center of the sheet without cracks and the sheet with cracks.

At the center of the sheet, the weakening of the texture (as shown in Figure 14) also has a certain influence on the mechanical properties of the sheet, leading to the simultaneous improvement of the plasticity and strength of the rolled sheet. The plasticity is from 8.4% to 10.2%, and the tensile strength is from 241 MPa to 259 MPa.

At the edge of the sheet, the sheet after conventional rolling has obvious cracks, as shown in Figure 16b, which is extremely unfavorable for the engineering application of magnesium alloy thin sheets. The edges of the sheet without cracks are smooth and almost no cracks occur, which can expand the industrial use of Mg alloy sheets. In addition to effectively suppressing the occurrence of cracks at the edge of the sheet, the performance of the edge of the Mg alloy rolled sheet without cracks is also greatly improved. As shown in Figure 16c, its plasticity is high and its strength can be reached. Compared with the center of the sheet, the mechanical properties are also greatly improved.

## 4. Conclusions

1. Crack propagation is due to the fact that conventional rolling will produce a large number of compressive twins and double twins under a large reduction in a single pass and cause a high degree of localization of the material strain, which becomes the nucleation point of microcracks, making the cracks spread along the twin lamellae and ends at the sheet where the dislocations accumulate. The structure of the sheet is unevenly distributed and exhibits hard orientation, which cannot coordinate strain and large plastic deformation, and the deformation energy and strain energy are released in the form of cracks.
2. In addition to the single-pass thickness reduction of 50%, the sheet without cracks is compressed along with TD during rolling, the large deformation causes various types of slip. Since the rolling temperature is 673 K, <a + c> slip that can coordinate the c-axis strain is activated and will cause lattice distortion. The prefabricated tensile twins can weaken the basal texture and soften the grain orientation, which is conducive to better coordination of strain and crack suppression.
3. A large number of dislocations are distributed in the matrix and the inside and outside the twins, making the twins shattered. The secondary twins are sheared and cannot develop into micro-cracks, avoiding the highly localized strain of the material. The deformation mechanism of dislocation shearing twins is an effective mechanism for crack inhibition, which can provide a theoretical basis for solving the problem of edge cracks in single-pass rolling with large thickness reduction.

## Figures and Tables

**Table 1:** Chemical composition of AZ31 magnesium alloy sheet.

Material	Al	Zn	Mn	Cu	Si	Ni	Fe	Mg
AZ31	2.99	1.03	0.2	0.01	0.08	0.002	0.003	Balance

**Table 2:** Rolling parameters for hot rolling of the magnesium alloys.

Sheet	Rolling Method	Rolling Temperature	Rolling Speed	Pass	Reduction per Pass
Sheet with cracks	Conventional rolling	673 K	10 rad/min	1	50%
Sheet without cracks	Limited width rolling	673 K	10 rad/min	1	50%

**Table 3:** Material properties of AZ31B used for Abaqus simulation.

Density	Elongation	Young’s Modulus	Poisson Ration	Tensile Strength	Yield Strength
1780 kg/m^3^	16.4%	52,479 MPa	0.34	260 MPa	87 MPa

## Data Availability

The data that support the findings of this study are available from the corresponding author upon reasonable request.
